# Identification of *TAZ* mutations in pediatric patients with cardiomyopathy by targeted next-generation sequencing in a Chinese cohort

**DOI:** 10.1186/s13023-016-0562-4

**Published:** 2017-02-10

**Authors:** Jian Wang, Ying Guo, Meirong Huang, Zhen Zhang, Junxue Zhu, Tingliang Liu, Lin Shi, Fen Li, Huimin Huang, Lijun Fu

**Affiliations:** 1 Research Division of Birth Defects, Institute of Pediatric Translational Medicine, Shanghai Children’s Medical Center, Shanghai Jiaotong University School of Medicine, Shanghai, 200127 People’s Republic of China; 2 Department of Cardiology, Shanghai Children’s Medical Center, Shanghai Jiao Tong University School of Medicine, 1678 Dongfang Road, Pudong, Shanghai, 200127 People’s Republic of China; 3 Research Division of cardiovascular disease, Institute of Pediatric Translational Medicine, Shanghai Children’s Medical Center, Shanghai Jiaotong University School of Medicine, Shanghai, 200127 People’s Republic of China; 4 Department of Cardiothoracic Surgery, Shanghai Children’s Medical Center, Shanghai Jiaotong University School of Medicine, Shanghai, 200127 People’s Republic of China

**Keywords:** Barth syndrome, *TAZ*, Cardiomyopathy, Targeted next generation sequencing

## Abstract

**Background:**

Barth syndrome (BTHS) is a rare X-linked recessive disease characterized by cardiomyopathy, neutropenia, skeletal myopathy and growth delay. Early diagnosis and appropriate treatment may improve the prognosis of this disease. The purpose of this study is to determine the role of targeted next-generation sequencing (NGS) in the early diagnosis of BTHS in children with cardiomyopathy.

**Methods:**

During the period between 2012 and 2015, a gene panel-based NGS approach was used to search for potentially disease-causing genetic variants in all patients referred to our institution with a clinical diagnosis of primary cardiomyopathy. NGS was performed using the Illumina sequencing system.

**Results:**

A total of 180 Chinese pediatric patients (114 males and 66 females) diagnosed with primary cardiomyopathy were enrolled in this study. *TAZ* mutations were identified in four of the male index patients, including two novel mutations (c.527A > G, p.H176R and c.134_136delinsCC, p.H45PfsX38). All four probands and two additional affected male family members were born at full term with a median birth weight of 2350 g (range, 2000–2850 g). The median age at diagnosis of cardiomyopathy was 3.0 months (range, 1.0–20.0 months). The baseline echocardiography revealed prominent dilation and trabeculations of the left ventricle with impaired systolic function in the six patients, four of which fulfilled the diagnostic criteria of left ventricular noncompaction. Other aspects of their clinical presentations included hypotonia (6/6), growth delay (6/6), neutropenia (3/6) and 3-methylglutaconic aciduria (4/5). Five patients died at a median age of 7.5 months (range, 7.0–12.0 months). The cause of death was heart failure associated with infection in three patients and cardiac arrhythmia in two patients. The remaining one patient survived beyond infancy but had fallen into a persistent vegetative state after suffering from cardiac arrest.

**Conclusions:**

This is the first report of systematic mutation screening of *TAZ* in a large cohort of pediatric patients with primary cardiomyopathy using the NGS approach. *TAZ* mutations were found in 4/114 (3.5%) male patients with primary cardiomyopathy. Our findings indicate that the inclusion of *TAZ* gene testing in cardiomyopathy genetic testing panels may contribute to the early diagnosis of BTHS.

## Background

Barth syndrome (BTHS; MIM 302060), first described in 1983, is a rare X-linked recessive disease caused by mutations in the *TAZ* gene located at Xq28 [1, 2]. It typically presents in males with cardiomyopathy, neutropenia, skeletal myopathy, growth delay and 3-methylglutaconic aciduria [3, 4]. Cardiomyopathy within the first year of life is the most common presentation and the primary cause of death in affected patients. However, in the absence of extracardiac features associated with BTHS (such as skeletal myopathy, neutropenia, growth retardation and 3-methylglutaconic aciduria), it may be difficult to distinguish BTHS from other infantile cardiomyopathies based on clinical presentations alone. Therefore, it seems likely that some patients with BTHS will remain undiagnosed unless mutation identification is obtained.

Genetic testing is now increasingly used as a means to confirm the specific diagnosis for patients with cardiomyopathy. However, genetic heterogeneity and phenotypic variability of the disease limit our ability to efficiently identify the underlying genetic cause using a candidate gene approach. Targeted next-generation sequencing (NGS) is a cost-effective approach for rapid and accurate detection of genetic mutations. Man et al. [5] recently employed NGS in two male siblings with isolated infantile dilated cardiomyopathy (DCM) and identified a hemizygous variant in the *TAZ* gene, suggesting that NGS may be used as a possible diagnostic strategy in BTHS. In the present study, we used a gene panel-based NGS approach to search for potentially disease-causing genetic variants in a large cohort of pediatric patients with cardiomyopathy of uncertain etiology. This led to the identification of *TAZ* mutations in 4/114 (3.5%) of the male index patients, including two novel mutations (c.527A > G, p.H176R and c.134_136delinsCC, p.H45PfsX38).

## Methods

### Patients and clinical evaluation

During the period between 2012 and 2015, all patients referred to our institution with a clinical diagnosis of primary cardiomyopathy were included in the study. All patients were evaluated by clinical history, physical examination, hematologic and biochemical laboratory analyses, electrocardiography (ECG), and echocardiography. Biochemical analysis of urine organic acids was investigated by gas chromatography-mass spectrometry according to standard methods.

In this study, neutropenia was defined by an absolute neutrophil count (ANC) below 1.5 × 10^9^/L. The corrected QT interval (QTc) was calculated from the 12-lead ECG using Bazett’s formula and a QTc of more than 440 milliseconds was considered as being prolonged. Echocardiography (2D, M- mode, and color Doppler) was used to evaluate the cardiac structure and function. DCM was defined as left ventricular ejection fraction (LVEF) <45% and left ventricular end-diastolic dimension (LVEDD) >2 standard deviations above the normal mean standardized to body surface area; hypertrophic cardiomyopathy (HCM) was defined as left ventricular posterior and/or septal wall thickness >2 standard deviations above the normal mean for body surface area in the absence of an identifiable hemodynamic cause [6]. The diagnosis of isolated left ventricular noncompaction (LVNC) was made by echocardiography on the basis of the criteria established by Jenni et al. [7], including: (1) a ratio of non-compacted to compacted layers of >2 measured in end-systole, (2) numerous prominent trabeculations and deep intertrabecular recesses filled with blood from the ventricular cavity as demonstrated by color Doppler, and (3) absence of associated cardiac abnormalities.

### Targeted panel-based next-generation sequencing

Peripheral blood was collected and genomic DNA was extracted according to standard procedures. Oligonucleotide-based target capture (Agilent SureSelect Target Enrichment System; Agilent, Santa Clara, California, United States) and subsequently NGS (Illumina HiSeq2500) were used to identify potential variants of 62 genes implicated in the causation of cardiomyopathy as described previously [8]. Alignment of sequence reads to reference human genome (Human 37.3, SNP135) was performed using the NextGENe® software (SoftGenetics, Stage College, Pennsylvania, USA). All single nucleotide variants and indels were saved as VCF format files, and uploaded to Ingenuity® Variant Analysis™ (Ingenuity Systems, Redwood City, California, USA) for variations filtering and interpretation. All the variations were classified according to the recommended method of the American College of Medical Genetics and Genomics. Pathogenic and potentially pathogenic mutations were confirmed by Sanger sequencing, where possible, validated by parental testing and segregation analysis. NM_000116.3 was used as the reference sequence for the coding regions of the *TAZ* gene. There was an approximate 4–6 week-period from laboratory receipt to report generation.

### Bioinformatic analysis of novel missense mutation

Phylogenetic conservation of the validated missense mutation was analyzed by the ClustalX program.

*In silico* predictions of the potential pathogenicity of a missense mutation was conducted by the following bioinformatics programs: MutationTaster (http://www.mutationtaster.org), Sorting Intolerant from Tolerant (SIFT) (http://sift.jcvi.org/), and PolyPhen-2 (http://genetics.bwh.harvard.edu/pph2/).

### Treatment and follow-up

All patients with a diagnosis of BTHS received standard heart failure medications and aspirin therapy, but no individual received granulocyte colony stimulating factor injections to prevent infection. All patients were followed up by either telephone interview or outpatient clinic visit. The primary outcome was death from any cause. The secondary outcome was cardiovascular event or severe infection that required medical supervision or hospitalization.

## Results

### Patients and molecular genetics

A total of 180 Chinese pediatric patients (114 males; 66 females) diagnosed with primary cardiomyopathy were enrolled in this study. Among the index cases, there were 64 patients (39 males and 25 females) with HCM, 72 patients (44 males and 28 females) with DCM, 27 patients (17 males and ten females) with LVNC and 17 patients (14 males and three females) with other types of cardiomyopathy. We performed targeted NGS on all these patients and identified *TAZ* mutations in four of 114 male patients, including three of the 17 male patients with LVNC and one of the 44 male patients with DCM. In contrast, no *TAZ* mutation was detected in the 66 female patients. The results were further validated by Sanger sequencing in probands and family members. The genetic features pertinent to the four probands and their family members are described below.

**Fig. 1:**
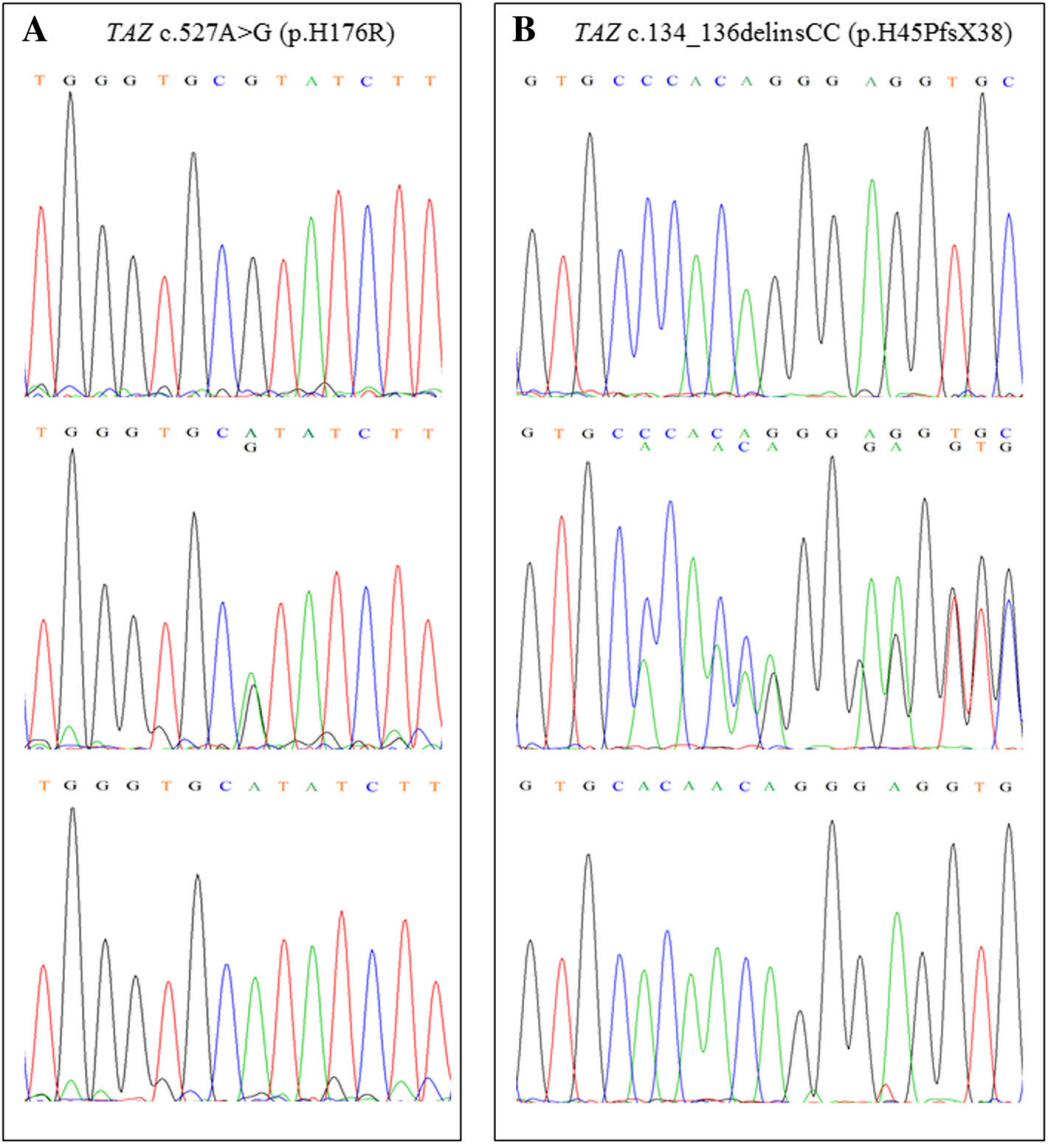
Sanger sequencing chromatograms. **a** Novel *TAZ* mutation c.527A > G (p.H176R) in proband 1: (*top*) Hemizygous mutation for the proband; (*middle*) Heterozygous mutation for the proband’s mother; (*bottom*) Hemizygous normal allele for the proband’s father. **b** Novel *TAZ* mutation c.134_136delinsCC (p.H45PfsX38) in proband 4: (*top*) Hemizygous mutation for the proband; (*middle*) Heterozygous mutation for the proband’s mother; (*bottom*) Hemizygous normal allele for the proband’s father

**Fig. 2:**
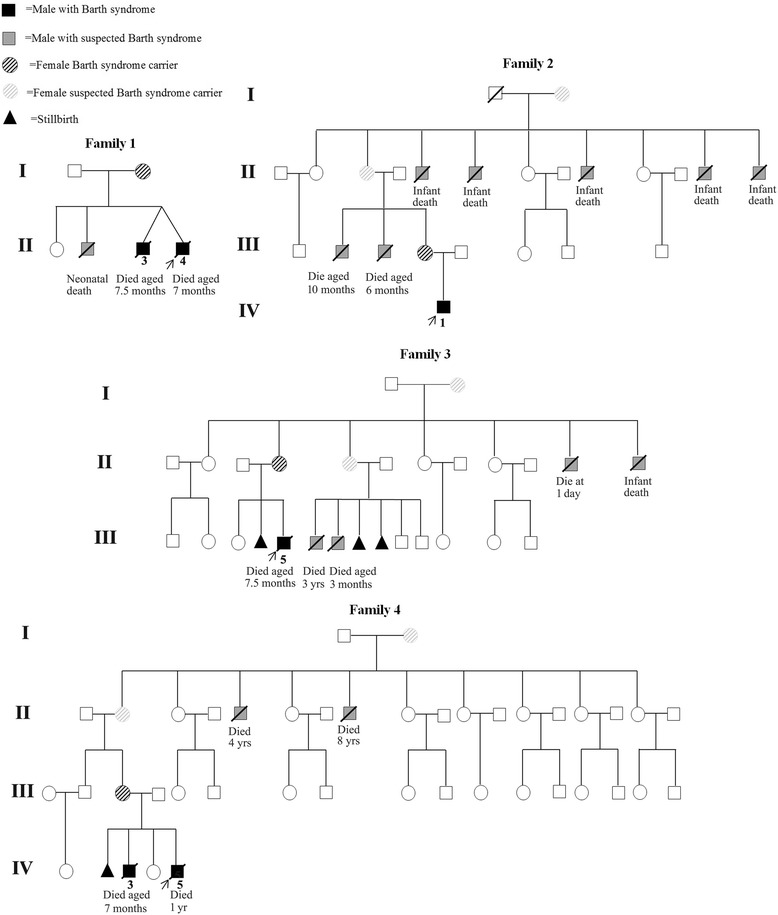
Pedigrees of four families discussed in detail in the paper. The proband is indicated by an *arrow*

A novel hemizygous missense variant c.527A > G (p.H176R) was identified in exon 6 of the *TAZ* gene in proband 1(BTHS1 in Table 1; II: four in Family 1 in Fig. 2). This variant was also identified in his affected twin brother (BTHS2 in Table 1; II: three in Family 1 in Fig. 2). Their unaffected mother was heterozygous for the same mutation, consistent with the X-linked recessive inheritance pattern (Fig. 1).

**Table 1:** Clinical and laboratory data of six Chinese patients with Barth syndrome

Patients	BTHS1	BTHS2	BTHS3	BTHS4	BTHS5	BTHS6
Gender	Male	Male	Male	Male	Male	Male
Birth weight (g)	2000	2400	2850	2300	2650	2300
First presentation	Pneumonia	Heart failure	Muscle weakness	Pneumonia	Heart failure	Pneumonia
Age of onset (months)	2.5	2.5	6.0	6.5	1.0	1.5
Age at diagnosis of cardiomyopathy	3.0	3.0	20.0	6.5	1.0	1.5
Growth retardation	+	+	+	+	+	+
Muscle hypotonia	+	+	+	+	+	+
Delayed motor	+	+	+	+	+	+
Echocardiogram						
LVEDD z-score at diagnosis	5.7	3.8	3.3	5.3	5.7	4.0
LVEF/LVSF at diagnosis (%)	45.6/22.1	36.2/16.7	40.1/19.1	36.8/17.3	40.1/18.9	43.0/20.0
Noncompaction/compaction (NC/C)	1.58	2.20	2.11	2.75	4.00	1.62
Electrocardiogram						
ST-T change	+	+	+	+	+	+
QT_C_ (milliseconds)	441	431	401	341	460	403
Neutropenia	-	-	+	+	+	-
Creatine kinase (range 55-170U/L)	81	60	62	23	46	43
3-methylglutaconic aciduria	+	+	+	+	-	Not detected
*TAZ* gene mutation	c.527A > G (p.H176R)	c.527A > G (p.H176R)	c.367C > T (p.R123X)	c.710_711delTG (p.V237AfsX73)	c.134_136delinsCC (p.H45PfsX38)	Not detected
Age at death (months)	7.0	7.5	Alive	7.5	12.0	7.0

*LVEDD* left ventricular end-diastolic dimension, *LVEF* left ventricular ejection fraction, *LVSF* left ventricular shortening fraction, *QT*_*C*_ corrected QT interval

A hemizygous variant c.367C > T (p.R123X) was detected in exon 4 of the *TAZ* gene in proband 2 (BTHS3 in Table 1; IV: one in Family 2 in Fig. 2), and a hemizygous frameshift variant c.710_711delTG (p.V237AfsX73) was identified in exon 10 of *TAZ* gene in proband 3 (BTHS4 in Table 1; III: five in Family 3 in Fig. 2). Both of the two mothers were obligate heterozygous carriers. These two variants have been previously reported to cause BTHS, indicating that the two variants were pathogenic [9].

A novel hemizygous frameshift variant c.134_136delinsCC (p.H45PfsX38) was detected in exon 2 of the *TAZ* gene in proband 4 (BTHS5 in Table 1; IV: five in Family 4 in Fig. 2). Sanger sequencing demonstrated the heterozygous status of his mother (Fig. 1). The proband’s elder brother (BTHS6 in Table 1; IV: three in Family 4 in Fig. 2) had clinical signs of BTHS and died of DCM at the age of 7 months, but blood samples were not available for mutation analysis.

### Confirmation of the likely pathogenicity of p.H176R

This variant c.527A > G (p.H176R) was absent in the database of dbSNP and 1000 Genomes, and not detected in 120 ethnicity-matched controls. Alignment of the amino acid sequence of tafazzin proteins showed that the histidine at position 176 was highly conserved across species (Table 2). This variant was predicted to be disease-causing with a score of one by MutationTaster, to be deleterious with a score of 0.000 by SIFT, and to be probably damaging with a score of 0.998 by PolyPhen-2.

**Table 2:** Multialignment of the amino acid sequence of tafazzin which surrounds the new p.H176R substitution identified in patient BTHS1

Orthologues	Amino acid sequence	Amino acid position
Human	L N H G D W V H I F P E G	169–181
Orangutan	L N H G D W V H I F P E G	139–151
Macaque	L N H G D W V H I F P E G	168–180
Mouse	L N H G D W V H I F P E G	139–151
Rat	L N H G D W V H I F P E G	139–151
Rabbit	L N H G D W V H I F P E G	139–151
Cow	L N H G D W V H I F P E G	139–151
Dog	L N H G D W V H I F P E G	167–179
Elephant	L N H G D W V H I F P E G	170–182
Fugu	L N R G D W V H I F P E G	162–174
Zebrafish	L N Q G D W V H I F P E G	139–151

### Family histories and clinical features

Family history was obtained in the four pedigrees. A high rate of premature male death was observed in the four pedigrees and a history of unexplained male fetal loss was observed in two pedigrees. This included one male neonatal death in family 1, seven male infant deaths in family 2, three male fetal stillbirths and four male neonate/infant/childhood deaths in family 3, one male fetal stillbirth and three male infant/childhood deaths in family 4. Taken together, there were four male fetal stillbirth and 15 premature male deaths in the four pedigrees. There were no losses of females. The pedigree charts are shown in Fig. 2.

In addition to the four probands, a thorough pedigree analysis led to the diagnosis of BTHS in two male family members, one (BTHS2 in Table 1; II: three in Family 1 in Fig. 2) with a confirmed *TAZ* mutation and the other one (BTHS6 in Table 1; IV: three in Family 4 in Fig. 2) with a presumptive diagnosis based on clinical signs of BTHS in a proven pedigree. The clinical features pertinent to the six patients are described below and summarized in Table 1.

All six patients were born at full term. The median birth weight was 2350 g (range, 2000–2850 g) and four patients had a birth weight below 2500 g. All six patients presented with symptoms prior to 1 year of age. The median age at presentation was 2.5 months (range, 1.0–6.5 months). Infection was the first symptoms in three patients. Cardiac failure was the first symptoms in two patients. Muscular weakness was the first symptoms in one patient.

**Fig. 3:**
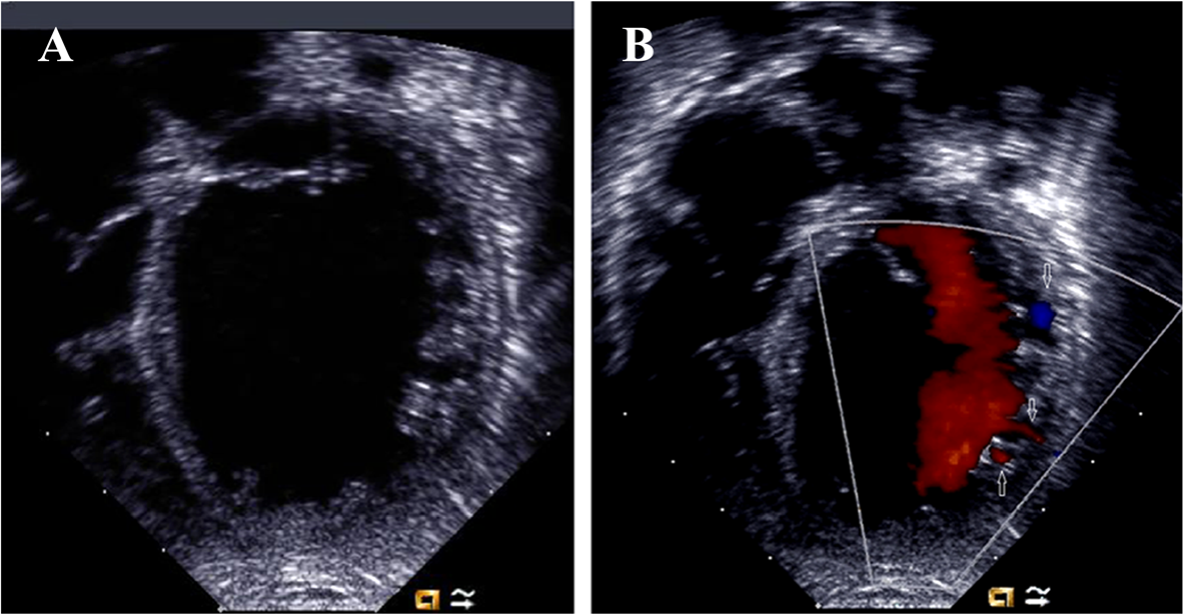
Echocardiogram (apical four-chamber view) of patient BTHS5 depicting LVNC with associated DCM phenotype. **a** Two-dimensional echocardiogram demonstrating the two-layer structure of noncompacted and compacted layers. **b** Color Doppler echocardiogram demonstrating flow within deep intertrabecular recesses (*arrow*) in continuity with the left ventricular cavity. LVNC = left ventricular noncompaction; DCM = dilated cardiomyopathy

The median age at diagnosis of cardiomyopathy was 3.0 months (range, 1.0–20.0 months). Five patients presented with symptoms of heart failure within the first year of life. The oldest patient of the cohort (BTHS3 in Table 1; IV: one in Family 2 in Fig. 2) presented with symptomatic cardiomyopathy at the age of 20 months. Baseline echocardiography revealed left ventricular dilation with impaired systolic function in the six patients. The mean LVEDD z-score was 4.6 ± 0.4, the mean LVEF was 40.3 ± 1.5%, and the mean left ventricular shortening fraction (LVSF) was 19.0 ± 0.8%. In addition, all the six patients had prominent trabeculations of the left ventricle on echocardiogram, 4 of which fulfilled the diagnostic criteria of LVNC. The remaining two patients also had prominent trabeculations of the left ventricle but did not meet the diagnostic criteria for LVNC. The region most frequently affected by noncompaction was the apex, followed by the posterior and lateral walls, mainly in the mid and apical segments (Fig. 3). Five patients also presented with dilatation of the left atrium.

The baseline ECG showed normal sinus rhythms in all six patients. Four patients had normal QRS duration, while the remaining two had intraventricular conduction delays. Ventricular repolarization abnormalities were seen in all six patients, predominantly ST flattening or T-wave inversion. The median QTc interval was 417 milliseconds (range 341–460 milliseconds), with one patient having prolonged QTc of 460 milliseconds. No supraventricular arrhythmias were detected in the six patients on admission. One patient had documented ventricular arrhythmias during hospitalization.

Failure to thrive was observed in all six patients on admission. Five patients were below—and one was at—the 3rd percentile in weight for their age. Likewise, five patients were below the 3rd percentile in height for their age, and one was at the 10th percentile in height for his age. Moreover, all six patients had muscle weakness and delayed developmental milestones, with normal serum creatine kinase levels. The oldest patient (BTHS3 in Table 1; IV: one in Family 2 in Fig. 2) of the cohort could not walk until the age of two.

Complete blood counts with differentials were measured in all six patients at original presentation. Neutropenia was documented in three patients and one of them had an ANC < 0.5 × 10^9^/L. However, none of the six patients had a low total white blood cell count, and normal hematocrit and platelets were observed in all individuals. Biochemical analysis of urine organic acids was performed in five patients, and four of them had a urinary 3-methylglutaconic level above the upper limit of normal.

### Survival

Five patients died at a median age of 7.5 months (range, 7.0–12.0 months). Patient BTHS1 (II: four in Family 1 in Fig. 2) and BTHS2 (II: three in Family 1 in Fig. 2) died of cardiac failure associated with high fever at the age of 7.0 and 7.5 months respectively. Patient BTHS4 (III: five in Family 3 in Fig. 2) failed to respond to aggressive treatment and died from repeated ventricular fibrillation when he was 7.5 months old. BTHS5 (IV: five in Family 4 in Fig. 2) exhibited chronic heart failure and intermittent neutropenia. He was hospitalized once for pneumonia and once for heart failure during 11-month follow-up. He died of cardiac failure associated with respiratory infection just beyond 1 year old. BTHS6 (IV: three in Family 4 in Fig. 2) was also hospitalized once for pneumonia and once for heart failure during 6-month follow-up, although he did not have documented neutropenia. He died of sudden cardiac arrest in home at the age of 7.0 months. The remaining one patient (BTHS3 in Table 1; IV: one in Family 2 in Fig. 2) showed an improvement in cardiac function with standard treatment and his LVSF had increased to 32.5%, but he had fallen into a coma after suffering from cardiac arrest when he was 33 months old. He was 38 months old at the last follow-up and showed normal cardiac function, but remained in a persistent vegetative state.

## Discussion

BTHS is thought to be an underdiagnosed cause of cardiomyopathy in children, though the involvement of the *TAZ* gene in common forms of cardiomyopathy is largely unknown [10]. To further evaluate the incidence of *TAZ* mutations in pediatric cardiomyopathy, we performed mutational analysis in a large cohort of unselected pediatric patients with primary cardiomyopathy and identified *TAZ* mutations in 4/114 (3.5%) male index patients. The prevalence of *TAZ* mutations in our cohort is similar to those from a comprehensive Australian study, which suggested that BTHS may constitute up to 4.8% of boys diagnosed with primary cardiomyopathy [11].

Various cardiac phenotypes have been described in patients with BTHS, such as DCM, isolated LVNC, HCM, or endocardial fibroelastosis. Transition between DCM and HCM phenotypes has also been reported in individuals with BTHS [3, 12]. DCM has been thought to be the most common cardiac phenotype caused by *TAZ* mutations. However, recent studies have indicated a high prevalence of LVNC in children with BTHS, either alone or in conjunction with other forms of cardiomyopathy [4]. In a large cohort study of BTHS, Spencer et al. [13] retrospectively reviewed echocardiographic images of 30 patients with BTHS and found that half of them had morphologic features of LVNC. In a French nationwide cohort study, LVNC was found in a third of patients with BTHS, although it might have been underestimated because of the retrospective nature of the study in which echocardiograms were not reviewed to search for prominent trabeculations [14]. In our present study, a total of six male children were diagnosed with BTHS and all of them presented with left ventricular dilation and impaired systolic function. Moreover, prominent left ventricular trabeculations were also observed in the six patients, 4 of which fulfilled the diagnostic criteria of LVNC. In contrast, no patient with a diagnosis of BTHS presented with HCM in our cohort. Our results suggested that LVNC with the DCM phenotype may be a rather common cardiac phenotype in BTHS, especially in infant-onset patients.

Cardiomyopathy may be the major clinical manifestation in patients with BTHS, but careful searching often reveals other signs of this multisystem disease as well as abnormal metabolites in blood or urine [15]. In our cohort, a total of six male patients from four unrelated families were diagnosed with BTHS. All individuals presented with documented heart failure and also a wide range of clinical features typically associated with BTHS such as neutropenia (3/6), delayed motor development (6/6), growth retardation (6/6) and 3-methylglutaconic aciduria (4/5). Furthermore, a high rate of premature male death was observed in the four pedigrees, which was consistent with an X-linked recessive pattern. In addition, a history of unexplained male fetal loss was observed in two pedigrees in our study, indicating that BTHS could lead to isolated or recurrent male fetal death as described by Steward et al. [16]. Our findings suggested that family history plays an important role in the evaluation of patients with possible BTHS and careful searching for extracardiac features associated with BTHS can contribute to the diagnosis of the disease.

BTHS is often fatal in infancy or early childhood as a result of heart failure and/or infections, which were observed in three patients in our cohort. A high prevalence of cardiac arrhythmia was also observed in our small series of young children with BTHS. Sudden cardiac death occurred in two patients during infancy, one from proven ventricular tachycardia with marked left ventricular dilation and very poor systolic function, and one from cardiac arrest with poor but stable cardiac function. Another patient suffered from cardiac arrest during a period of apparent well-being when he was 33 months old with mild left ventricular dilation and normal systolic function. These findings suggested that the risk of cardiac arrhythmias may be independent of the degree of left ventricular dilation or dysfunction, which is consistent with the findings by Spencer et al. [17].

BTHS is caused by mutations in the *TAZ* gene located at Xq28. Encoded by the *TAZ* gene, tafazzin is a phospholipid transacylase located in the mitochondrial inner membrane and plays an important role in the remodeling of cardiolipin [18]. Tafazzin harbors five putative acyltransferase motifs and an integral interfacial membrane anchor, all of which are highly conserved and strongly related to the mutations observed in patients with BTHS [19]. Up to date, more than 160 different mutations have been reported in the Human Tafazzin (*TAZ*) Gene Mutation and Variation Database (http://www.barthsyndrome.org/), including missense, nonsense, splicing, and frameshift mutations. In the present study, four different mutations were identified, two of which were novel. The novel frameshift mutation, c.134_136delinsCC (p.H45PfsX38), was predicted to introduce a premature stop codon at position 83, while the full-length of tafazzin protein is 292 residues long. Premature stop codons usually lead to the degradation of the affected mRNA transcripts by a surveillance pathway termed nonsense-mediated mRNA decay [20], resulting in the loss-of-function of the affected gene. Notably, a frameshift mutation, c.171delA (p.G58AfsX25), predicted to truncate at the same stop codon, has already been described in BTHS patients elsewhere [21], providing additional evidence to support the causative role of our newly identified frameshift mutation. The pathogenicity of the other novel mutation c.527A > G (p.H176R) is suggested by numerous lines of evidence: (i) This variant c.527A > G (p.H176R) was absent in current databases of dbSNP and 1000 Genomes, and in 120 ethnicity-matched controls. (ii) This histidine residue is located in the putative motif C of the tafazzin protein and is extremely conserved during evolution, implying its functional importance. (iii) Multiple well-known computer algorithms, such as MutationTaster, SIFT, and PolyPhen-2, consistently predict that this novel mutation is deleterious and displays high disease-causing potential. (iv) Family pedigree also indicates that this mutation co-segregates with disease phenotypes.

BTHS is a multisystem disorder with highly variable clinical presentations. Early diagnosis and appropriate treatment may improve the prognosis. Unfortunately, the diagnosis of this disease is often delayed or missed because the characteristic symptoms of BHTS may vary in severity and are not consistently present in every patient [10]. BTHS is also known as 3-methylglutaconic aciduria type II, but 3-methylglutaconic aciduria is not consistently present in every patient with BTHS, as observed in only one patient in this study. Neutropenia is a classical characteristic of BTHS and represents an important clue for BTHS diagnosis [14]. However, the absence of neutropenia in three of the six patients at diagnosis in our study suggests that a normal ANC count in male infants with cardiomyopathy does not exclude BTHS. In a large cohort study of BTHS, ninety percent of patients had a clinical history of cardiomyopathy diagnosed at an average age of 5.5 months, but the genetic diagnosis of BTHS was not made until an average age of 4.6 years [13]. The use of NGS has recently been reported as a possible diagnostic strategy in BTHS, but not yet been widely implemented [22]. The present study demonstrates that target NGS provides a novel, rapid, simple, and highly sensitive screening method for the early detection of this disease.

## Conclusions

BTHS should be considered in male children with primary cardiomyopathy, especially in male infancy with LVNC. The inclusion of *TAZ* gene in cardiomyopathy genetic diagnostic panels may contribute to early diagnosis of BTHS.

## Abbreviations

ANC: Absolute neutrophil count
BTHS: Barth syndrome
DCM: Dilated cardiomyopathy
ECG: Electrocardiography
HCM: Hypertrophic cardiomyopathy
LVEDD: Left ventricular end-diastolic dimension
LVEF: Left ventricular ejection fraction
LVNC: Left ventricular noncompaction
LVSF: Left ventricular shortening fraction
NGS: Next-generation sequencing
QTc: corrected QT interval
SIFT: Sorting intolerant from tolerant

## References

[1] Barth PG, Scholte HR, Berden JA, Van der Klei-Van Moorsel JM, Luyt-Houwen IE, Van’t Veer-Korthof ET (1983). An X-linked mitochondrial disease affecting cardiac muscle, skeletal muscle and neutrophil leucocytes. J Neurol Sci.

[2] Bione S, D’Adamo P, Maestrini E, Gedeon AK, Bolhuis PA, Toniolo D (1996). A novel X-linked gene, G4.5 is responsible for Barth syndrome. Nat Genet.

[3] Clarke SL, Bowron A, Gonzalez IL, Groves SJ, Newbury-Ecob R, Clayton N (2013). Barth syndrome. Orphanet J Rare Dis.

[4] Jefferies JL (2013). Barth syndrome. Am J Med Genet C Semin Med Genet.

[5] Man E, Lafferty KA, Funke BH, Lun KS, Chan SY, Chau AK, et al. NGS identifies TAZ mutation in a family with X-linked dilated cardiomyopathy. BMJ Case Rep. 2013. doi: 10.1136/bcr-2012-007529. 10.1136/bcr-2012-007529 PMC3604426 23345479

[6] Grenier MA, Osganian SK, Cox GF, Towbin JA, Colan SD, Lurie PR (2000). Design and implementation of the North American Pediatric Cardiomyopathy Registry. Am Heart J.

[7] Jenni R, Oechslin E, Schneider J, Attenhofer Jost C, Kaufmann PA (2001). Echocardiographic and pathoanatomical characteristics of isolated left ventricular non-compaction: a step towards classification as a distinct cardiomyopathy. Heart.

[8] Fu L, Luo S, Cai S, Hong W, Guo Y, Wu J (2016). Identification of LAMP2 mutations in early-onset danon disease with hypertrophic cardiomyopathy by targeted next-generation sequencing. Am J Cardiol.

[9] Ferri L, Donati MA, Funghini S, Malvagia S, Catarzi S, Lugli L (2013). New clinical and molecular insights on Barth syndrome. Orphanet J Rare Dis.

[10] Cantlay AM, Shokrollahi K, Allen JT, Lunt PW, Newbury-Ecob RA, Steward CG (1999). Genetic analysis of the G4.5 gene in families with suspected Barth syndrome. J Pediatr.

[11] Nugent AW, Daubeney PE, Chondros P, Carlin JB, Cheung M, Wilkinson LC (2003). National Australian Childhood Cardiomyopathy Study. The epidemiology of childhood cardiomyopathy in Australia. N Engl J Med.

[12] Hanke SP, Gardner AB, Lombardi JP, Manning PB, Nelson DP, Towbin JA (2012). Left ventricular noncompaction cardiomyopathy in Barth syndrome: an example of an undulating cardiac phenotype necessitating mechanical circulatory support as a bridge to transplantation. Pediatr Cardiol.

[13] Spencer CT, Bryant RM, Day J, Gonzalez IL, Colan SD, Thompson WR (2006). Cardiac and clinical phenotype in Barth syndrome. Pediatrics.

[14] Rigaud C, Lebre AS, Touraine R, Beaupain B, Ottolenghi C, Chabli A (2013). Natural history of Barth syndrome: a national cohort study of 22 patients. Orphanet J Rare Dis.

[15] Vernon HJ, Sandlers Y, McClellan R, Kelley RI (2014). Clinical laboratory studies in Barth Syndrome. Mol Genet Metab.

[16] Steward CG, Newbury-Ecob RA, Hastings R, Smithson SF, Tsai-Goodman B, Quarrell OW (2010). Barth syndrome: an X-linked cause of fetal cardiomyopathy and stillbirth. Prenat Diagn.

[17] Spencer CT, Byrne BJ, Gewitz MH, Wechsler SB, Kao AC, Gerstenfeld EP (2005). Ventricular arrhythmia in the X-linked cardiomyopathy Barth syndrome. Pediatr Cardiol.

[18] Saric A, Andreau K, Armand AS, Moller IM, Petit PX (2015). Barth Syndrome: From Mitochondrial Dysfunctions Associated with Aberrant Production of Reactive Oxygen Species to Pluripotent Stem Cell Studies. Front Genet.

[19] Karkucinska-Wieckowska A, Trubicka J, Werner B, Kokoszynska K, Pajdowska M, Pronicki M (2013). Left ventricular noncompaction (LVNC) and low mitochondrial membrane potential are specific for Barth syndrome. J Inherit Metab Dis.

[20] Lykke-Andersen S, Jensen TH (2015). Nonsense-mediated mRNA decay: an intricate machinery that shapes transcriptomes. Nat Rev Mol Cell Biol.

[21] Kirwin SM, Manolakos A, Barnett SS, Gonzalez IL (2014). Tafazzin splice variants and mutations in Barth syndrome. Mol Genet Metab.

[22] Brión M, de Castro López MJ, Santori M, Pérez Muñuzuri A, López Abel B, Baña Souto AM (2016). Prospective and Retrospective Diagnosis of Barth Syndrome Aided by Next-Generation Sequencing. Am J Clin Pathol.

